# More Bang for Your Buck: Best-Practice Recommendations for Designing, Implementing, and Evaluating Job Creation Studies

**DOI:** 10.1007/s13132-023-01199-8

**Published:** 2023-03-14

**Authors:** Paloma Bernal-Turnes, Ricardo Ernst

**Affiliations:** 1 Business Economics Dept., Rey Juan Carlos University, Paseo de los Artilleros s/n, 28032 Madrid, Spain; 2 Global Logistics Research Program, McDonough School of Business, Georgetown University, 37th and O St. NW, Washington, DC 20057 USA; 3 The World Bank, 2121 Pennsylvania Ave. NW, Washington, DC 20433 USA

**Keywords:** Randomized experiments, Job creation, Finance, Matching grants, Theory of Change, Impact evaluation

## Abstract

This paper discusses the application of robust experimental research methodologies that help to provide a better understanding of the mechanisms of the Theory of Change, for which training programs and/or matching grants improve job creation in micro, small, and medium-sized enterprises (MSMEs and SMEs). The literature on both interventions, such as training and matching grants, recognizes methodological flaws that hamper achieving enough statistical evidence to test the aforementioned Theory of Change. A better understanding of the interventions and the mechanisms to create jobs has become critical to ensure the resurgence of the global economy after the COVID-19 pandemic and to face the threat of the upcoming industrial revolution. This paper proposes seven methodological meliorations in impact evaluation that will help to set improvements alongside the full process of a project: designing superior policies and programs, implementing projects, supporting the finer assessment of interventions, and establishing the subsequent advancement of science in testing solutions for job creation.

## Introduction

Impact evaluation is the tool to support evidence-based policy making because it serves as a foundation for managing projects with a better assessment of the performance of development programs and policies. The evidence derived from a strong methodology in impact evaluation is crucial when challenging and developing new theories, thus creating knowledge about what, how, and in which extension a certain intervention contributes to a specific goal. Indeed, the attribution of what and how it can generate the desired outcomes (which is the definition of the Theory of Change) is a question of paramount significance in both economic and the entire social science; this is due to the fact that the peerless contribution of an impact evaluation is to determine and estimate causal relationships between policies and outcomes. Thus, a greater knowledge of causal effects could help to solve the crucial problem of job destruction after the pandemic of COVID-19, if we could only find out what, how, and in which extension a specific intervention creates jobs.

The need for improving our knowledge of causal effects has been claimed by many researchers in social science (Shadish et al., 2002; Uy et al., 2010; Miller and Tsang, 2011; Allen et al., 2014; Podsakoff et al., 2014). The required methodologies for understanding the direction and nature of causal relationships (Spector, 1981; Grant and Wall, 2009; Aguinis and Bradley, 2014) are the true field of experimental methods which consist of randomized and quasi-randomized experiments (the latter are also called “quasi-experiments”). We are warned that methodological advancement tends to disseminate slowly (Aguinis et al., 2009), and it has been argued that management scholars do not know how to conduct field experiments (Borsboom et al., 2009; Highhouse, 2009). Thus, building a robust methodology in impact evaluation will provide us with the evidence that an action may distinctly, unambiguously, and almost unerringly cause a certain outcome of interest and the means by which the results occur (Lopez-Acevedo and Tan, 2011; Gertler et al., 2016). However, experiments in social science are scarce, as can be seen in the literature revisions in top journals (Podsakoff and Dalton, 1987; Scandura and Williams, 2000; Austin et al., 2002; Grant and Wall, 2009; Cravo and Piza, 2016; Buba et al. 2020; Dvouletý et al., 2021). Additionally, even when research is based on experiments, the analysis of concrete and attributable findings from impact evaluation on a specific topic is not always an easy task for practitioners, donors, or researchers, due to the lack of comparability of the studies.

The complexity to compare outcomes in impact evaluations in social science comes from a wide variety of sources. First, one of the difficulties to extract valid information from impact evaluation cases comes from the different perspectives that stakeholders could have about any project. Donors and policymakers are more likely driven by the outcome of a project, the “what to achieve,” such as job creation, because they are involved in providing solutions for a certain problem, which could be the reduction of the high unemployment rate. In contrast, other stakeholders involved in impact evaluation projects, such as practitioners and economists, are more likely to focus their attention on tools and inputs to achieve those outcomes, the “how to achieve it.” Thus, the provision of the service becomes the essence of the monitoring mechanisms. Continuing with this example, the “how” to achieve job creation could be done by a wide variety of policies and services, such as the provision of the application of new technology, basic education, matching grants, or capacity building. We argue that it is important to reconcile the different perspectives that link outcomes and inputs, theory and practice, and policies and facts, in the belief that they are interrelated. We hope that this paper encourages scientists and practitioners to build bridges between problems and solutions for which impact evaluation projects are designed.

Second, when research is based on experiments, the analysis of concrete and attributable findings from impact evaluation requires transparency in reporting results, as this will help assemble evidence from programs. Meta-analysis of research literature, longitudinal studies, and Bayesian analysis facilitate the understanding of the generalization of causal effects with different participants, time, treatment variations, settings, and research methodologies (Hedges and Olkin, 1980, 1985; Hedges, 1987; Howard et al., 2000; West and Thoemmes, 2010). We highlight that there is not yet solid evidence that the creation of jobs in SMEs is attributable to matching grants (Hristova and Coste, 2016; Piza et al., 2016; Cravo and Piza, 2016). Indeed, the revision of the literature on the effects of the provision of finance with matching grants on job creation provides a too wide range of conclusions that go from inconclusive results (Tan and Lopez-Acevedo, 2005; Bruhn and Love, 2012; McKenzie et al., 2015), negative effects (Rijkers et al., 2010; Karlan et al., 2015; Fiala, 2018), inaccurate effects (Lopez-Acevedo and Tinajero, 2010), and non-effects (Bruhn et al., 2012). More transparency when reporting will avoid conflicting results.

This paper exposes a set of methodological improvements that define a rational process to increase accuracy in impact evaluation, followed by the advancement of science. These methodological improvements are the methodological dreams that we would like to see come true in the years to come, with the hope to get conclusive findings and advance the practical and theoretical knowledge about job creation in SMEs. By reviewing the literature about the intervention training and matching grants, we have identified some relevant aspects that are not sufficiently addressed to properly test the Theory of Change. The following are the impact evaluation improvements we dream of for the methodological research flaws:

1. the time frame to test theories;
2. the sample size;
3. the effect size and power;
4. the descriptive analysis;
5. the budget allocation;
6. the generality of results; and
7. the outcomes.

## Advancement in Theoretical Progress

Acknowledging that the advancement of science relies on the accumulation of knowledge, “social science” (such as Economics or Business Management in the topic of job creation) versus “hard science” (such as Medicine, Physics, or Astronomy) faces several threats in this empirical comparison (Borsboom et al., 2009; Highhouse, 2009). The acid test for the theoretical advancement is the replicability of results (Hedges, 1987), but specific aspects in social science obstruct theoretical progress (Pfeffer, 1993) and the consensus on scientific paradigms (Davis, 2010). In Economics and Business Management stand out the lack of standardized tools and unstructured methodological procedures (Ketchen et al., 2008; Edwards, 2008; Hitt et al., 2004; Aguinis and Edwards, 2014) which deter the performance of experiments as it is similarly done in hard science for ensuring, unerringly, the attribution of results (Cravo and Piza, 2016). More specifically, in the realm of job creation in SMEs within the Theory of Change, the comparison of social science experiments is difficult because there is ambiguity and imprecision when standardizing core concepts and tool definitions (Cravo and Piza, 2016; Grimm and Paffhausen, 2015), such as the concept of SME; not all environments and legal frameworks have accepted the consensus on the threshold of 250 employees (Ayyagari et al., 2007; Cravo et al., 2012), which limits consistency in the results and the development of theories.

Replicability in social science also requires isolating the effects of pervasive interactions (Cronbach 1975), historical and cultural influences (Gergen, 1973, 1982), unstandardized measurements (Kruskal, 1978), and blurry indirect and moderated effects (King et al., 2012; Bernal and Ernst, 2015, 2016; Eden et al., 2015) to achieve more precise and more unerringly attributable outcomes for SME interventions.

Additionally, researchers suggest that the advancement of science needs more than the rejection of the null hypothesis, even if the statistical power is acceptable (Meehl, 1978; Bezeau and Graves, 2001). Solving big economic and social dilemmas of our era, such as job creation, requires different data collection techniques from different sources of data, multi-method statistical analysis, and novel uses of technologies (Gregorie et al., 2010). The integration of new technologies in true field experiments is opening new opportunities to test theories and measure concepts of social science with more accuracy (Aguinis and Lawal, 2012; Hsu et al., 2017). Economics should rely more on complementary formats for doing research, integrating different analysis techniques such as descriptive analysis, graphical methods, geospatial analysis, and meta- and Bayesian analysis.

### Time Frame to Test Outcomes

The first methodological melioration in impact evaluation is linked to the length of the experiment; it should be based on the nature of the program and always be embedded in the literature on the specific intervention and the outcome. It has been suggested that the length of the program is one of the main threats of previous studies to sufficiently capture the impacts searched (Cravo and Piza, 2016). The right length of the program depends on the magnitude of the expected outcomes, the trend of which could follow a linear or a non-linear pattern. Indeed, the evaluation of the full effect of some programs, such as training (Mano et al., 2012) or technology diffusion (Hall and Khan, 2003), requires both a prudent time to elapse between intervention and outcomes (because short-run effects are limited) and several rounds of follow-up surveys that build panel data to observe the efficiency of a policy. Multiple waves of measurement will allow us to shape the curve-fitting relationship between intervention and outcome. For example, the learning curve of new skills or the productivity curve of technology adoption is impossible to observe with only two-time measurements (pre- and post-treatment), and it could be even worse, if the time that elapses between the baseline and the end line of the program is too short, the outcomes should be imperceptible, and therefore, not statistically significant.

Our methodological dream for the coming years is to see more publications in impact evaluations that test the results of training and/or matching grants in several waves to capture curve effects (Mano et al., 2012; Histrova and Coste, 2016). More specifically, the desirable timeframe to observe results for matching grant projects to create jobs is approximately 2 years (Rijkers et al., 2010; McKenzie et al., 2015), while training interventions have a shorter-run impact on performance and productivity (McKenzie et al., 2015); however, it could be imperceptible in the short-run. Additionally, it is important to contextualize the stage of entrepreneurial experience when inferring causality (Hsu et al., 2017). It has been tested that entrepreneurship activity unfolds over time and incurs differently in the outcomes (Shane, 2003; Shane and Venkataraman, 2000; Haltiwanger et al., 2013).

### Sample Size

The second recommendation for doing more robust impact evaluations is the need to perform more accurate calculations of a minimum sample size, which is needed to test the impact of any intervention. The size of the sample has a tremendous influence on research costs, but it also affects the quality of the study. Unfortunately, it is possible to read that sample sizes that are too small are the reason for having inconclusive results in some project interventions (Cravo and Piza, 2016). Too small a sample size can lead to the paradox of obtaining very different means in the treatment and the control, while that difference is not statistically significant. In other words, differences in means between treatment and control groups are happening by coincidence, instead of happening in every sample extracted from the population. The paradoxical failure of using a sample size that is not large enough drives us to either useless interventions to achieve desired outcomes or, what could be even worse, to reach real outcomes that are not observable from an effective intervention, whatever could be the significance level of acceptance of the hypothesis (Khandker et al., 2010; Gertler et al., 2016). Acknowledging the problem caused by small sample sizes in specific impact evaluations, conclusions such as a much higher survival rate of those MSMEs whose entrepreneurs receive training (Mano et al., 2012), or a much higher increase in the business performance of those MSMEs that receive consultancy services, could not be taken into consideration for being statistically insignificant (Bruhn et al., 2010).

The sample size is also linked with the significance level (called alpha), or the Type I error rate, which is the probability of rejecting H_0_ (null hypothesis) when it is actually true. The smaller the Type I error rate, the larger the sample required for the same power. Power in statistics identifies how reliable the results obtained are, which is linked to the Type I error rate. In other words, the larger the sample size, the higher the Type I error.

The calculation of the sample size is linked with the calculation of the statistical power, which is higher as the size of the treatment and control group are equal (Cohen, 1994; Khandker et al., 2010; Gertler et al., 2016). Besides the sample size calculation to achieve a specified standard error and a certain probability of statistical significance (Gelman and Hill, 2006), the sample size must be big enough to easily identify similar means and standard deviations in the control and treatment groups in the baseline. The effect size is the metric that calculates the distance between the control and treatment groups in means and standard deviations. We should be able to detect the smallest effect size possible because it will help to directly attribute the outcomes of the intervention in the endline.

Impact evaluations, when well-constructed, build two subsets of the sample with the following criteria: (1) the treatment group must be a reliable and a fair representation of the full population, (2) the control group must be the mirror of the treatment group in terms of its main characteristics, and (3) replications of the study will bring similar results. These requirements to build the treatment and the control group (analysis ex-ante) and rigorous empirical methods to analyze the causal effects (analysis ex-post) will allow to determine the outcomes that are uniquely, distinctly, and directly attributable to the matching grants (Khandker et al., 2010; Gertler et al., 2016). Thus, SMEs after being randomly reached out with open access communication campaigns to apply for the financial support, which ensures equal opportunities to participate in the intervention, must be assigned the treatment through randomization.

Consequently, a comparison group will be constructed from the applicant pool of those firms that did not receive any grant, but entities are similar to the first sample on observed pre-intervention characteristics. Linked to the decision of the method to be used to build the comparison group and estimated the counterfactual, we need to calculate the sample size.

Our methodological dream for the years to come is to see more publications on impact evaluations. These can provide sample size calculations for the building of the control and treatment groups and for performing the main analysis of the experiment tailored by the type of statistical methodology applied and the number of variables (and estimations) considered.

### Power and Effect Size

Most literature in impact evaluation is focused on reporting the statistical significance of the validity of a null hypothesis, as it is reported in any other scientific area of knowledge. However, power analysis very often is underreported in social science or scarcely done with a transparent approach (McKenzie et al., 2015).

The power analysis provides a unique piece of information in impact evaluations: The implied probability of making an error of estimation. The power of a statistical test of a null hypothesis is the probability of making the correct decision, that is, the probability of being right in the rejection of the null hypothesis if the alternative hypothesis is true (Cohen, 1988; Cohn and Becker, 2003). When power is low, the more likely it is to make a Type II error, which is the probability of rejecting the null hypothesis when the alternative hypothesis is true. In the pre-treatment phase of any experiment, even with significantly similar means in the treatment and control groups, with low power, there is a higher risk to assume that both groups have similar means when it is false. In other words, there is a high risk to get inconclusive results from an experiment in which the control group differs significantly from the treatment group. Under this scenario, it is important to realize that with Type II errors, it will be quite unlikely to determine that the outcomes are uniquely, unerringly, and fully attributable to the intervention executed. Then, the Theory of Change will be wrongly texted in the post-intervention analysis under high Type II errors in the pre-treatment analysis.

An example to illustrate that low power could lead us to a wrong conclusion is the case in which the treatment group has a higher job creation rate than the control group, but instead, the test of means differences (effect size) in the pre-treatment stage leads us to think that both groups are significantly similar. In this case, if the power of the test is very low, then, we will be more likely to wrongly assume that the intervention to facilitate access to finance (executed only in the treatment group) is undoubtedly the reason for having a significantly higher job creation rate than the control group, which is false. Only with the power test can we check the probability of being wrong in the conclusion. When we increase power, we reduce Type II error, and we are more likely able to say that our findings are robust, because firms under equal conditions (the job creation means are significantly equal in a pre-treatment test) create more jobs when they have access to the finance intervention (during the post-treatment test). The key point here is that, under low-powered testing, effects statistically significant tend to vary greatly from different samples, producing patterns of apparent contradictions in the published literature (Maxwell, 2004; Cohn and Becker, 2003), reducing theoretical precision that impedes the generalization of policies and programs. In other words, seriously underpowered impact evaluations are useless, while, in turn, the increase in the statistical power builds coherence in the literature and advances scientific knowledge (Maxwell, 2004).

Literature shows the persistence of lack of distinction in the power analysis done in each experiment (Cohen, 1994; Saris and Satorra, 1993; Satorra and Saris, 1985). Indeed, any single study should include multiple power analyses (Maxwell, 2004) such as the incremental explanatory power analysis (Biscotti and D’Amico, 2019). First, the experimental design will require testing the power in building the sample size to determine the number of subjects needed in the study to detect an effect of a given size (Cohen, 1994). Additionally, power analysis must be done to design the main statistical analysis tailored to the research methodology applied: the larger the number of explanatory variables, the larger the sample size is required (Maxwell, 2004).

Power analysis and sample size calculations could be conducted using Cohen’s tables (Cohen, 1994) or software such as G*Power (Faul et al., 2009), SAMPLE POWER (SPSS, 2017), and R (Green and MacLeod, 2016). Calculations of statistical power depend on the alpha significance, the sample size, and the effect size (Bezeau and Graves, 2001). The most common way to solve underpowered analysis is by increasing the sample size, which easily raises the cost of the experiment. However, other three less costly actions allow for the increase of statistical power in the post-treatment tests. First, the use of more advanced applied methodological techniques, such as multilevel analysis (Kozlowski and Klein, 2000) and the addition of covariates (Satorra and Saris, 1985; Judd and McCelland, 1989; Maxwell et al., 2018). Second, the formulation of simple null hypotheses, rather than formulating complex null hypotheses (McClelland, 1997). Third, in case we wish to test a dichotomic outcome, the use of a more efficient allocation of observations that maximize the variance with half of the sample in the two extreme values (½ 0 0 ½), instead of using a standard normally distributed across the mean (¼ ¼ ¼ ¼) (Mead, 1988; Atkinson and Donev, 1992; McClelland, 1997).

Additionally, in the power analysis, effect size should be reported too. Effect size measures the distance between the treatment and the comparison group (Bezeau and Graves, 2001). If the research is able to detect small effect sizes of a treatment, it will lead to a better detection of the causal effects between interventions and outcomes. Cohen’s (1988) conventional definitions of small, medium, and large effect sizes for each statistic measure are usually the most commonly used tool (Mone et al., 1996).

Cohen (1988, 1992), Hunter and Schmidt (1990), Lykken (1968), Rosenthal (1991), and Mone et al. (1996) highlighted two advantages of reporting and evaluating effect size in research. First, the effect size reports the magnitude of the phenomenon in the population (Mone et al., 1996), and then, the comparability of studies. Second, reporting effect sizes increases the replicability of research streams and the comparability of studies with meta- and Bayesian analysis (Maddock and Rossi, 2001; Hedges and Olkin, 1980, 1985). Notice that the problem of comparing studies with unequal effect-sized groups is the invalid comparison of chi-squares that have different degrees of freedom (Hedges, 1987).

There are, at least, three ways to increase effect size, besides increasing sample size. First, the use of more advanced methodological techniques, such as multilevel analysis (Kozlowski and Klein, 2000). Second, the use of more reliable measurements using the formulae suggested by Schmidt et al. (1976) or Schmitt and Klimoski (1991) for increasing the validity of estimations to increase the statistical power and reduce the need for larger samples (Sawyer and Ball, 1981; Schmidt et al., 1976; Sutcliffe, 1980). Last of all, the use of statistical methods of Cascio and Zedeck (1983) for reducing the alpha level and then increasing the power.

Our methodological dream for the years to come is to see more publications in impact evaluations that provide and report the analysis of power and effect size. It is a tenet of good practice if both calculations are made while designing the impact evaluation. We encourage researchers to use advanced statistical methodologies, besides sample size, to increase statistical power and the capacity to detect small effect sizes.

### Descriptive Analysis

Impact evaluation is not an exception in Economics for not carrying out a deep descriptive analysis of what the data shows in both the treatment and control groups before conducting inferential statistics. Traditionally, studies that use impact evaluation select the control group in terms of similarity of the central tendency measures, mainly mean or median, with the treatment group. The assumption of normality for the treatment and control group should not be assumed, and instead, it must be analyzed in the baseline, especially for experiments with small sample sizes. Once the normal distribution is tested in the treatment and control group, the larger the standard deviation in the control group, the higher the probability of making error type II in our conclusions. Then, the analysis of the standard deviation is critical for increasing internal validity and power, besides a bigger sample size. Our methodological dream about the descriptive analysis is to incorporate the analysis of the measure of variability with the standard deviation in the control group. We hope to see more publications with experimental and quasi-experimental designs that provide complete descriptive analysis and test the similarity of means and standard deviations in the treatment and control groups in the pre-treatment phase.

## Incorporating Realistic Challenges During Implementation

Previous methodological studies about impact evaluation are not naive in assuming changes that could arise during the implementation, and indeed, they reveal that evaluation designs quite often are not implemented as initially stated (Gertler et al., 2016). This section is created on the belief that some research methodological improvements for succeeding in inferring causation require close cooperation among practitioners and researchers. Said cooperation could help to

- eradicate biased samples,
- identify context-specific phenomena,
- alert of events that disrupt outcomes (such as changes in legal frameworks),
- identify and collect cofounder variables, and
- build a common understanding that enriches the perspective of the research.

The combination of experience on the ground, the strong cooperation with local stakeholders and donors, and the advice from researchers and economists could help to identify the right research design and execution (Grant and Wall, 2009; Rynes and Bartunek, 2017). Indeed, impact evaluation not only advances knowledge but also improves project implementation itself, as it helps to allocate resources and to increase the accountability of the project (Legovini et al., 2015).

### Budget Allocation

Monitoring and evaluation systems allow for the implementation of programs and interventions with transparency and accountability for the sake of an effective budget management. When more than one intervention is executed at the same time, researchers should design and report the scientific findings with a clear distinction of the following three aspects: (a) samples (from beneficiaries and control groups), (b) interventions, and (c) budgets, to study the effectiveness of each intervention with a proper and precise estimation of the causal effect to achieve a specific outcome (Lopez-Acevedo and Tinajero, 2010).

Clear accountability of interventions allows showing the results achieved in measurable outcomes. These could be translated into a convertible currency, personnel, or time length, which provides a better understanding of the findings and facilitates the attraction of investors by explaining, for instance, the dollars needed to create each job, the personnel needed to provide training to increase a certain amount of revenue, or the number of months needed to create each job position. This improvement in budgeting interventions helps researchers to generalize the study. Additionally, researchers shed light, not only on the effect size (in our examples in dollars or months), but on the importance of the “cause size” in comparing experiments (Highhouse, 2009).

Our methodological dream for the years to come is to see more publications on impact evaluations that facilitate independent budget allocation of each intervention and samples separately and, additionally, specific budget allocation for each interaction of programs when they exist.

### Generalization of Results

The advancement in knowledge in answering a cause-effect question requires the generalization of results achieving broader effectiveness and scalability under two different angles: on the one hand, by testing alternative programs to achieve the same outcomes, and on the other, by testing a causal effect with the Theory of Change applied in the distinctiveness of research settings (Cook and Campbell, 1979).

The first angle of analysis consists of testing the effectiveness of a series of alternative programs in a particular setting, timeframe, and population, which contributes to the generalization of results, as it validates the status of the theory when, separately, different interventions are applied in the same scenario to achieve the same outcome, such as creating jobs either by providing matching grants (Hristova and Coste, 2016; Piza et al., 2016; Cravo and Piza, 2016) or by facilitating access to external markets (Rossignol and Salmon, 2016). Then, the body of evidence became more robust concerning the bundle of benefits, threats, and spillovers when reaching a specific goal under different interventions in the same environment (Highhouse, 2009; Rijkers et al., 2010). This approach not only advances science but also brings very valuable information to policymakers, because it provides information about the policy that is more adequate to solve a problem.

The other angle to generalize results consists of testing the properties of a certain theory that should be applied in multiple settings or audiences, such as testing a specific mechanism to create jobs, in both peaceful versus conflicted and violent environments. Under this angle, the alternative explanations of the effects have been isolated, and the remaining attributable effects of the intervention are tested separately in both scenarios. Only then, the generalization of results could be possible, because the analysis provides the information on the effectiveness of a certain intervention with and without the different circumstances that affect the scenario, recognizing the minimum and maximum effect of an intervention: either the environment is peaceful or conflicted and violent. Research shows that the mechanism to create jobs in conflict and violent environments requires the analysis of the effects of uncertainty on entrepreneurs’ decisions for taking risks (Knight, 1921; McMullen and Shepherd, 2006; Ralston, 2014; Mallet and Slater, 2016); in other words, it should require control for uncertainty to do the variables consistent across conditions (Hsu et al., 2014, 2017; Ashta et al., 2021). The studies made by McKelvie et al. (2011), Koudstaal et al. (2015), and Holm et al. (2013) are experiments that include the analysis of entrepreneurs’ decisions and actions process under conditions of uncertainty. Indeed, job creation in conflict and violent environments is a way of building social cohesion, allowing the transformation of informal into formal businesses, and improving the inclusivity of ex-combatants and potential insurgents (Ralston, 2014).

Under both angles of building knowledge, the reduction of the sources of bias increases the external validity and then increases the generalizability of the results. The methodological ameliorations that reduce the sources of bias can be achieved by correctly identifying and clearly explaining the following items: (1) the selection of the population of interest to the research question asked, (2) the attributes of the context that influence the sample or subsets of the sample, (3) the active or passive nature of the individuals analyzed (Hsu et al., 2017), (4) the research question embedded in the field of science, and (5) the adoption of the statistical methodology tailored to the research question and the program’s operational characteristics (Khandker et al., 2010; Gertler et al., 2016).

Additionally, from both angles, the advancement of science in job creation could be trapped in not having enough proof to distinguish between null findings that result from low power (Cohn and Becker, 2003) and null findings that reflect a genuine absence of effect size that results from the wide length of the confident interval (Howard et al., 2000). This dilemma is not yet solved in experiments in SMEs, since its literature scarcely reports confidence intervals (McKenzie et al., 2016) and power analysis as has been discussed before. Besides the contribution to solving this dilemma about null findings, reporting confidence intervals around the mean provides three main additional advantages. First, confidence intervals provide information about the precision of the estimate because it implies the value of a hypothesis test, which is the zero value within the interval. Thereby, the precise estimation of the null hypothesis of no difference could not be rejected in a tiny degree of error, generally stated at an alpha level of .05 (McCallum et al., 1996; Howard et al., 2000). Second, confidence intervals provide further information beyond “yes-or-no” outcomes (the yes or no non-zero population effects), as the smaller the confidence intervals, the higher the precision and the better the estimation of effect size (Cohen, 1994; Cohn and Becker, 2003). Finally, confidence intervals facilitate the theoretical interpretation of its central point (Bezeau and Graves, 2001).

Literature also reflects the concern that the reliance on tests of statistical significance contributes to the poorer theoretical and empirical cumulativeness of knowledge in social science that hampers the generalization of results (Meehl, 1978; Hedges, 1987). Meta-analysis of studies and Bayesian analysis, however, can increase the likelihood of detecting population effects (Hedges and Olkin, 1985; Hedges, 1987; Hunter and Schmidt, 1990; Maddock and Rossi, 2001; Cohn and Becker, 2003; Aguinis and Edwards, 2014), and they focus on the magnitude of a treatment effect, such as the magnitude of jobs creation through matching grants.

Our methodological dream for finding an answer to the questions about job-creating interventions in an unbiased way is to encourage authors to state the findings in terms of the magnitude of the effects with their confidence intervals in order to better answer this theoretical conundrum (Cohen, 1994; Aguinis and Edwards, 2014). Then, the comparison and assembly of evidence through confidence intervals is linked with the use of meta-analysis, Bayesian analysis, and longitudinal techniques and could solve the problems created by low statistical power in individual studies (Hunter and Schmidt, 1990; Cohn and Becker, 2003).

### Outcomes to Test the Theory of Change

Randomized experiments are not always the gold standard for research design in social science for the advancement in the knowledge of the Theory of Change (Campbell and Stanley, 1966; Cook and Campbell, 1979; Grant and Wall, 2009). Quasi-experiments are also fundamental for building and generalizing strong theories in social science (Dubin, 1976; Whetten 1989; Grant and Wall, 2009). Indeed, quasi-experiments ensure the rigorous construction of boundary conditions under a certain treatment, which is more or less likely to exert a particular pattern of effects (Johns, 2006). Thus, it is the ideal analysis for interventions that affect certain groups differently within the sample (Khandker et al., 2010; Gertler et al., 2016). Additionally, quasi-experiments are for unethical problems to randomize the treatment and the control group (Khandker et al., 2010; Gertler et al., 2016). For these two reasons, quasi-experiments require judicious research choices and rigorous methodologies that expand internal and external validity.

Quasi-experiments have traditionally been seen as a silver medal for testing causal effects (King et al., 2012), and the gold one was designated for randomized experiments (Shadish et al., 2002; King et al., 2012). Other authors, however, defend the advantages of quasi-experiments over randomized experiments in terms of implementation, validity, and testing (Campbell and Stanley, 1966; Cook and Campbell, 1979). Richer discussions have also emerged about experiments (Highhouse, 2009; Bullock et al., 2010; Aguinis and Lawal, 2012; King et al., 2012; Eden, 2017). Indeed, generalization in social science requires careful attention by cofounders (McKelvie et al., 2011; Holm et al., 2013; Hsu et al., 2014, 2017; Koudstaal et al., 2015), sampling stimuli, and strengthening manipulations (Highhouse, 2009) to undoubtedly understand causal effects.

Estimating the impact of the treatment in quasi-experiments depends on constructing a valid counterfactual group that parallels the SME beneficiary group in all respects except for participation in the intervention under evaluation. For this purpose, Propensity Score Matching (PSM) will be used to create statistically equivalent counterfactuals to the treatment group. The evaluation question, expressed in the following expression, can be simplified as estimating the average treatment effect by taking the difference between the expected outcomes of the treatment and comparison groups:

$$ATE=\left[E\left( Yi(1)|T=1\right)\right]-\left[E\left( Yi(0)|T=0\right)\right]$$

where “ATE” is the average treatment effect and “Yi” is the outcome of the ith SME unit.

The outcomes for the treated and comparison SME units are

$$\mathrm{Yi}\left(1\right)=\mathrm{Yi}\left(\mathrm T\;=\;1\right)\;\mathrm{for}\;\mathrm{the}\;\mathrm{matching}\;\mathrm{grant}\;\mathrm{treatment}\;\mathrm T\;\left(\mathrm{treated}\right)$$

$$\mathrm{Yi}\left(0\right)\;=\;\mathrm{Yi}\left(\mathrm T=0\right)\;\mathrm{for}\;\mathrm{the}\;\mathrm{matching}\;\mathrm{grant}\;\mathrm{treatment}\;\mathrm T\;\left(\mathrm{comparison}\right)$$

PSM addresses the problem of a missing counterfactual: The fundamental idea of the PSM approach is that for each unit in the treatment group and in the pool of non-selected firms, the probability of treatment (propensity score) is computed based on observed characteristics. Background covariates of selection (e.g., firm size, age, number of employees, sector) into treatment are converted into this single scalar propensity score, thereby reducing multidimensionality. The score, ranging from 0 to 1, is the SMEs probability of receiving treatment conditional on observed covariates. This quasi-experimental approach ensures that the average characteristics of the treatment and comparison groups are similar, which is a necessary condition to obtain unbiased estimates. The impact of grants on beneficiaries can be estimated by comparing the average outcomes of a treatment group and the average outcomes of a statistically matched subgroup of firms, the match being based on observed characteristics available in the data at hand.

Applying a matching evaluation design would translate into the following broad steps:

1. Estimate the propensity score using either the probit or logit model. P [x] = P[ T = 1 | x], i.e., the probability of receiving the treatment [T = 1] given a set of observed characteristics.
2. Choose an appropriate matching method to match the estimated propensity scores of treated SME units to untreated SME units—Methods such as nearest neighbor, radius, stratification, kernel, caliper, and others can be used. We propose using the 2:1 nearest neighbor technique, based on the principle of minimizing the absolute difference between the estimated propensity scores for the control and treatment groups.
3. Assess the quality of the matching by checking for common support and balance, and as a result, restricting the sample to units for which common support appears in the propensity score distribution.
4. For each treatment unit, locate a subgroup of comparison group units that have similar propensity scores.
5. Compare the outcomes for the treatment units and their matched comparison units. The difference in average outcomes for these two subgroups is the measure of the impact that can be attributed to the program for that particular treated observation
6. The mean of these individual impacts yields the estimated average treatment effect or ATE (i.e., difference in outcomes between the participants and matched non-recipients).

Our methodological dream is to see more publications that coherently build the realm of knowledge with either quasi- and randomized experiments to build the Theory of Change that can explain the causal effect of job creation or quasi-experiments to understand the circumstances, contexts, and groups that exert differently in terms of job creation.

## Conclusions

The main originality of this paper is to identify the main methodological meliorations that support evidence-based policy making for the creation of jobs. The surge in the demand for a better assessment of the performance of job creation programs and policies has become critical in providing prosperity. This paper should drive practitioners and researchers to benefit from methodological improvements for inferring causation in financial support to SMEs to create jobs. The revision of previous studies shows evidence that firm-level experiments focused on the impacts of interventions on job creation are complex to infer positive direct effects (Buba et al., 2020; Dvouletý et al., 2021). More often than desired, the provision of financial support to SMEs provoked unseeking results on job destruction among the beneficiaries of the interventions (Fiala, 2018; Karlan et al., 2015; Rijkers et al., 2010). In this regard, although implementation and monitoring are at the heart of evidence-based policy making, the impact evaluation should also use a core set of statistical tools to ensure that the outcomes on job creation are uniquely, unerringly, and fully attributable to the intervention executed. Our hope is that by linking implementation best practice recommendations to the methodological meliorations in impact evaluation, we will be able to catalyze further research in the area and, ultimately, to support more efficient policies that bring prosperity through job creation.

## Theoretical Implications

Our study identifies methodological meliorations that ensure robust statistical evidence and accurate assessment of the true impact of financial support interventions in SMEs. Most of the methodological improvements suggested in this paper obey the concern about how fundamental pre-intervention analysis, such as sampling, matching, and time framework, is in causation (Rubin, 1974, 2007, 2008; Cook and Steiner, 2010). This thought was very nicely stated by Campbell (1969a, 1969b), Rubin (2007, 2008), and Cook and Steiner (2010), who said that it is not possible to put right with statistics what has been wrong by design.

Our study suggests that the decision of applying randomized versus quasi-experiments has some trade-offs. Randomization maximizes internal validity but, in contrast, undermines external validity, while quasi-experiments in many organizational settings could provide superior external validity with good levels of internal validity (Grant and Wall, 2009; Campbell and Stanley, 1966).

This paper shows that, apart from increasing sample size, the reduction of the standard deviation in the control group brings higher power, as well as the subsequent reduction of Type II error in the analysis. But, in contrast, just by increasing the sample size, it boosts the Type I error. For this reason, power at the level of .8 is acceptable in experiments to solve this trade-off and to more confidently detect and reject false null hypotheses (Martínez, 2022). Only in two situations are unpowered studies justified (Halpem et al., 2002): (1) in interventions that are aimed to solve situations that affect either odd cases or a very limited number of individuals; (2) in early-phase trials for defining better ulterior purposes of an intervention.

In our methodological dreams, impact evaluations always report effect sizes (besides power analysis too). For the determination of the control and treatment group in the matching pair analysis, a small effect size is recommended, while for the main statistical analysis that tests the treatment effects on outcomes, a medium effect size seems an appropriate standard to do the power analyses (Cohen, 1962, Bezeau and Graves, 2001). However, it is relevant to highlight that important findings were detected when an effect size was really small in the main statistical analysis of the experiment, as it happened with the finding that headache pills reduce heart attacks. This experiment was done with a very large sample size of 22,000 individuals and a very small effect size (.0022). Social science literature, and specifically economic impact evaluation studies, should reveal power analysis more often and link and reveal a certain level of power (at least .8) with the smallest effect size possible (Mone et al., 1996).

Similar information on effect sizes is provided by confidence intervals (Cohen, 1994; Bezeau and Graves, 2001). Confidence intervals reveal the status of the null hypotheses and the non-nil null hypotheses and facilitate the generalization of knowledge because it allows for the comparison of results. Based on the advantages of calculating confidence intervals, why do authors not report them? Findings suggested that confidence intervals are the thermometer of imprecise findings (Cohen, 1994; Howard et al., 2000). Any underpowered research is more likely to incorrectly accept false null hypotheses that contain erroneous conclusions about the hypotheses tested in impact evaluation (Mone et al., 1996; Tversky and Kahneman, 1971).

Erroneous conclusions hamper the advancement of science because it provides conflicting results as well as conclusions (Smith, 1977; Grant and Wall, 2009). The statistical explanation for conflicting results in impact evaluation is named Type III error. Type III errors occur when the null hypothesis is false and is rejected for being the direction of the true population contrary to the direction of the observed difference (Kaiser, 1960; Leventhal and Huynn, 1996; Highhouse, 2009). The problem is that it is difficult to detect Type III errors in social science. It is possible to reduce Type III errors when conflicting impacts are tested if sample stimuli occur. A recommendation for avoiding conflicting results in social science experiments is to control the mechanisms in which control and treatment groups could share information or attitudes that affect the outcomes. Conflicting results could appear in non-linear causal effects, for which the analysis of mediation and moderation effects could provide a wider picture of the intensity, and even the direction, of the effects that a treatment exerts on the outcomes (Bullock et al., 2010; King et al., 2012; Eden et al., 2015). Another solution to avoid selection bias and get the true impact of financial support to SMEs is to test the robustness of results applying Rosenbaum’s (2002) bounding approach (Alemu and Ganewo, 2022).

But also richer discussions have emerged about experiments (Highhouse, 2009; Bullock et al., 2010; Aguinis and Lawal, 2012; King et al., 2012; Eden, 2017). Indeed, generalization in social science requires more careful attention to cofounders (McKelvie et al., 2011; Holm et al., 2013; Hsu et al., 2014, 2017; Koudstaal et al., 2015) and sampling stimuli (Highhouse, 2009) to undoubtedly understand causal effects.

A clear budget allocation is a ¨should¨ that, besides the advantages of accountability, helps researchers to show findings in a tangible and appealing way for the comparison of studies.

One of our methodological dreams also reflects a concern about the timing of the experiment, in which the length of the project should be adapted to the environmental challenges. Experiments also allow testing the Theory of Change with temporal progression (Lopez-Acevedo and Tinajero, 2010). Time series analysis provides stronger empirical evidence and provides the course, the strength, and the direction of the outcomes.

In summary, if robust research methodologies are as important as we believe it to be, research that yields insight into the mechanisms behind their development and the strategic choices on which they rest could make an important scientist contributions on a wide variety of topics.

## Managerial Implications

Impact evaluations are needed to inform policymakers on a range of decisions, from curtailing inefficient programs, to scaling up interventions that work, to adjusting program benefits, to selecting among various program alternatives. Business support interventions focused on SMEs are crucial since it has been tested that SMEs generate the majority of employment in developed and developing countries (Ayyagari et al., 2011). Unfortunatelly, SMEs encounter astounding low productivity performance and overcome barriers to grow (Mead and Liedholm, 1998, Alemu and Ganewo, 2022). These two aspects support high-priority policies that target SMEs especially in environments that face several constraints including no access to finance, shortage of equipment, low productivity, outdated technology, and lack of skilled labor forces. Matching grants sounds that could address these constraints in situations where formal financial institutions are not willing to take any exposure beyond very basic financial services such as deposit collection, payments, and remittances. The market failure is thus evident from the credit crunch aggravated by the post-COVID scenario, especially in less developed economies and fragile and conflict environments.

Beside these market imperfections to allocate financial resources, the analysis of results shows that there are two managerial aspects underlying suboptimal allocation of inputs that have implications to design more effective policies: (1) behavioral biases—such as misperception of returns associated with a given business practice, lack of motivation to adopt better production process (Gibbons and Henderson, 2012), and cultural barriers to access to formal financial services (Alemu and Ganewo, 2022); and (2) organizational barriers that prevent firms from adopting new technologies (Atkin et al., 2017) and using inputs optimally. In this light, the policy implication of these findings is that, besides easier access to finance, interventions should be aimed at training managers and employees on the use of new technologies and better managerial practices to increase the formal access to credit and public services, which are key to SMEs’ growth.

The complexity of interventions to create jobs requires close collaboration among scientists, stakeholders, and practitioners, since the impact is closely related to how research is organized and the intervention is implemented (Taverdet-Popiolek, 2022). In this regard, researchers scarcely provide information about the process evaluation. Process evaluations focus on how a program is implemented and operates, assessing whether it conforms to its original design and documenting barriers on its development and operation. Evidence from process evaluations can complement impact evaluation results and provide a more complete picture of program performance, shedding light on how processes are functioning such as risks and barriers to accomplish the planning. This is particularly important in building the sample: While project beneficiaries can be held accountable to respond to the survey, response rates are likely to be lower among non-beneficiaries. Increasing response rates among non-beneficiaries will involve creating sufficient incentives for them to participate in the baseline and ex-post surveys.

## Future Research

We propose the following research questions to be addressed in future studies more deeply in order to get more robust results to better answer the challenging question of how to create more jobs:

- Do researchers report more transparently the results of the experiments (such as randomization of participants, matching technique, effect size, power analysis, confidence intervals, time effects, budget allocation)?
- Do researchers and practitioners work cooperatively to design the research, monitor the implementation of the intervention, and do the process evaluation?
- Are the effects of matching grants consistent across business sizes, business ages, entrepreneurs ages, gender, sectors, urban vs. rural business location, peaceful and stable vs. fragile and conflict environments, technology usage, and number of business partners and diverse business network?
- Are the samples and the budget allocation clearly defined for each research question and intervention?
- Are the counterfactual and spillover effects sufficiently evaluated?
- Is there any potential nonlinearity of policy impact on job creation?

All these research questions could reduce the inefficiency of policies in order to boost the expected outcomes in job creation.

## Data Availability

Not applicable.
